# Asymmetric Safety Corridors for Free-Hand S2-Alar-Iliac Screw Placement: Quantifying Direction-Specific Tolerance Around Patient-Specific Optimal Trajectories

**DOI:** 10.3390/jcm15124495

**Published:** 2026-06-10

**Authors:** Se Jun Park, Dong Kyu Kim, Sun Joon Yoo, Hyun Jun Jang, Bong Ju Moon, Jeong Yoon Park, Jun Jae Shin, Sung Uk Kuh, Dong Kyu Chin, Keun Su Kim, Kyung Hyun Kim

**Affiliations:** 1 Department of Neurosurgery, Yongin Severance Hospital, Yonsei University College of Medicine, Yongin 16995, Republic of Korea; dannypsj@yuhs.ac (S.J.P.);; 2 Department of Neurosurgery, Gangnam Severance Hospital, Yonsei University College of Medicine, Seoul 03722, Republic of Korea

**Keywords:** S2-alar-iliac screw, sacropelvic fixation, pelvic tilt, screw trajectory, safety margin, spinal deformity

## Abstract

**Background/Objectives:** Optimal trajectories for S2-alar-iliac (S2AI) screw placement have been widely studied; however, in fluoroscopy-assisted free-hand techniques, exact reproduction is rarely achievable. This study aimed to quantify direction-specific safety margins around patient-specific optimal trajectories and to determine their relationship with pelvic parameters. **Methods:** We retrospectively analyzed patients who underwent S2AI screw fixation with available preoperative and postoperative CT imaging. Pelvic parameters, including pelvic tilt (PT), sacral slope (SS), and pelvic incidence (PI), were measured. Optimal transverse and sagittal screw angles were determined using CT-based planning. Postoperative CT was used to assess actual screw trajectories and cortical violations. Direction-specific generalized estimating equation models were used to evaluate associations between trajectory deviation and screw malposition. Receiver operating characteristic (ROC) analysis was performed to determine cutoff values for safe deviation. **Results:** A total of 62 patients (105 screws) were included in axial analysis and 41 patients (76 screws) in sagittal analysis. PT and PI showed significant inverse correlations with both optimal transverse and sagittal angles (all *p* < 0.001). Greater lateral and medial deviations were significantly associated with corresponding cortical violations (OR 2.33, 95% CI 1.51–3.59; and OR 2.10, 95% CI 1.40–3.15 per degree, respectively; both *p* < 0.001). Inferior deviation was significantly associated with violation in the sagittal plane (OR 1.39, 95% CI 1.18–1.65 per degree; *p* < 0.001), whereas superior deviation was not significant. ROC analysis demonstrated asymmetric safety margins: 1.5° lateral (AUC = 0.972), 8.1° medial (AUC = 0.965), and 18.5° inferior (AUC = 0.897). **Conclusions:** S2AI screw placement may be conceptualized as a tolerance-based process centered on a patient-specific optimal trajectory. Safety margins are direction-dependent and asymmetric, with a narrow tolerance for lateral deviation. These findings provide practical guidance for intraoperative trajectory adjustment in free-hand techniques.

## 1. Introduction

Secure sacropelvic fixation is essential for achieving durable fusion in long spinal constructs, yet it remains technically demanding for surgeons [1,2] due to its distinctive anatomical and biomechanical characteristics [3,4]. Among the available techniques, S2-alar-iliac (S2AI) screw fixation, first introduced by Sponseller [5] and Kebaish [6], has become a widespread procedure for sacropelvic fixation because of its advantages over conventional iliac screw insertion [7,8,9]. Proper insertion is vital to avoid injuring major vessels, sacral nerves, and sacroiliac joints [10]. O’Brien et al. reported a 15% rate of iliac cortical perforation during free-hand fluoroscopy-assisted S2AI screw placement in a cadaveric study [11].

Previous studies have emphasized identifying ideal trajectories for S2AI screw insertion [5,6,12,13,14]. Recent research has further refined these ideals by demonstrating that pelvic parameters, specifically pelvic tilt (PT) and pelvic incidence (PI), significantly influence the optimal trajectory [15,16]. However, these prior studies have focused almost exclusively on defining theoretical “perfect” angles rather than addressing how such values translate to actual surgical practice.

In practice, surgeons rely on fluoroscopic landmarks, and some degree of deviation from the optimal trajectory is inevitable. Furthermore, the allowable deviation range in certain directions remains incompletely characterized in the literature, which may affect intraoperative decision-making.

The purpose of this study was to quantify direction-specific tolerance margins around patient-specific optimal S2AI trajectories. We hypothesized that these safety margins would be asymmetric and clinically meaningful for intraoperative decision-making. 

## 2. Materials and Methods

### 2.1. Study Design and Patient Population

This retrospective study included adult patients (≥18 years) who underwent S2-alar-iliac (S2AI) screw fixation as part of posterior spinal instrumentation at a single tertiary referral center between May 2013 and December 2023. Indications for surgery included adult spinal deformity and degenerative spinal conditions requiring sacropelvic fixation.

Patients were excluded if they had a history of prior hip surgery, spinal or pelvic tumors, spinal infection, acute trauma involving the pelvis or sacrum, or congenital pelvic abnormalities. Patients were also excluded if preoperative or postoperative radiological imaging was inadequate for reliable measurement of pelvic parameters or screw trajectories.

This study was approved by the Institutional Review Board of our institution, and the requirement for informed consent was waived due to the retrospective design.

### 2.2. Surgical Technique

All procedures were performed by two spine surgeons, each with more than five years of experience in complex spinal deformity surgery and S2AI screw placement. The screws were inserted using a free-hand technique under fluoroscopic guidance. The entry point was defined as the midpoint between the lateral margins of the S1 and S2 dorsal foramina. In the sagittal plane, the screw was directed toward the anterior inferior iliac spine (AIIS), whereas in the axial plane the trajectory was aimed toward the narrowest portion of the ilium, consistent with previously described techniques [16]. Implanted S2AI screws were 8.5 mm in diameter and 70–80 mm in length, with final screw selection based on patient anatomy and surgeon preference. Commercially available pedicle screw systems (Solera^®^, Medtronic, Minneapolis, MN, USA; or Innoverse^®^, CGBio, Seongnam, Republic of Korea) were used.

### 2.3. Radiological Assessments

Preoperative pelvic parameters, including pelvic tilt (PT), sacral slope (SS), and pelvic incidence (PI), were measured using CT scout images according to established definitions [17]. CT-based measurements were used rather than standing radiographs, because screw trajectory planning was performed based on preoperative CT anatomy, and screw insertion occurs in the prone position where pelvic orientation differs from standing alignment. Preoperative and postoperative CT scans were obtained using a slice thickness of 2 mm and were reviewed using multiplanar reconstruction images. All radiographic measurements were performed using the institutional picture archiving and communication system (PACS) measurement tools.

Two key trajectory angles were measured using preoperative CT imaging: transverse lateral angle, defined as the angle between the planned screw trajectory and the sagittal midline in the axial plane, and sagittal caudal angle, defined as the angle between the planned screw trajectory and the horizontal reference line in the sagittal plane.

The optimal transverse lateral angle (OTA) and optimal sagittal caudal angle (OSA) were defined as the longest possible intraosseous pathway without cortical violation throughout the sacral and iliac corridor [16]. During trajectory planning, both medial/lateral and superior/inferior cortices were simultaneously considered to ensure a fully intraosseous pathway. The planned trajectory was directed toward the anterior inferior iliac spine (AIIS), consistent with previously described S2AI techniques [16]. The same anatomical entry point, defined as the midpoint between the S1 and S2 dorsal foramina, was used during both preoperative CT-based trajectory planning. Screw diameter was standardized to 8.5 mm during planning, while screw length of 70–80 mm was individualized according to the maximal achievable intraosseous length.

Postoperative CT scans were used to assess actual screw trajectories. Angle deviation was defined as the signed difference between the actual postoperative screw trajectory and the preoperatively determined optimal trajectory. This allowed directional analysis of medial, lateral, superior, and inferior deviations.

Screw placement was classified based on cortical violation observed on postoperative CT. Cortical violation was defined as any radiographically visible breach of the cortical boundary by any portion of the screw on postoperative CT images. In the axial plane, malposition was categorized as medial or lateral violation, while in the sagittal plane it was categorized as superior or inferior violation.

Postoperative CT scans obtained in earlier cases did not consistently include the distal iliac region up to the femoral heads due to limited scan range. As a result, accurate assessment of screw tip position and cortical violation was not feasible in these cases, and they were excluded from analyses requiring full trajectory evaluation. These limitations predominantly affected sagittal plane assessment, resulting in a reduced sample size for sagittal analysis.

All measurements were independently performed by two spine surgeons, and the mean values were used for statistical analysis. Interobserver reliability was assessed using the intraclass correlation coefficient (ICC) based on a two-way random-effects model for absolute agreement.

### 2.4. Statistical Analysis

Continuous variables are presented as mean ± standard deviation, and categorical variables as counts and percentages. Pearson correlation coefficients were calculated to evaluate the relationships between pelvic parameters (PT, SS, PI) and optimal trajectory angles (OTA and OSA).

Because some patients contributed bilateral screws, observations were not fully independent; therefore, generalized estimating equation (GEE) models clustered by patient ID were used to account for within-patient correlation. GEE models using exchangeable working correlation structure were used to evaluate the association between deviation magnitude in each direction and the corresponding cortical violation. In each model, the magnitude of deviation toward the direction of interest (e.g., medial deviation for medial violation) was included as a continuous predictor.

Receiver operating characteristic (ROC) curve analysis was performed to determine cutoff values of trajectory deviation associated with screw malposition in both axial and sagittal planes. The optimal cutoff values representing the safety margins from the optimal trajectory were determined based on the maximal Youden index. The area under the curve (AUC) and 95% confidence intervals (CI) were calculated.

A *p*-value < 0.05 was considered statistically significant. All statistical analyses were performed using SPSS software (version 28.0; IBM Corp., Chicago, IL, USA).

## 3. Results

### 3.1. Patient Demographics

**Figure 1:**
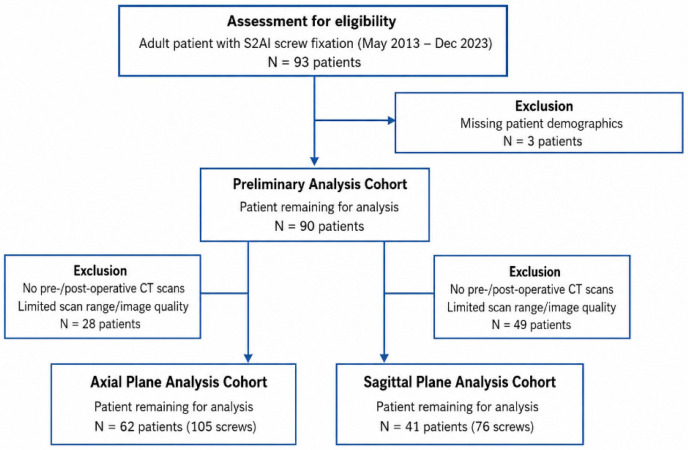
Flowchart of patient and screw selection.

A total of 93 adult patients underwent S2AI screw fixation between May 2013 and December 2023. Three patients were excluded because demographic data were unavailable. Of the remaining 90 patients, 28 were excluded from the axial plane analysis and 49 from the sagittal plane analysis because of unavailable imaging or insufficient CT scan range/image quality. Consequently, 62 patients (105 screws) were included in the axial plane analysis and 41 patients (76 screws) in the sagittal plane analysis (Figure 1). Among the 62 patients included in the axial analysis, 43 had bilateral S2AI screws and 19 had unilateral screws. Among the 41 patients included in the sagittal analysis, 35 had bilateral screws and 6 had unilateral screws.

Baseline characteristics of the included screws are summarized in Table 1. No significant differences in age, body mass index (BMI), or bone mineral density (BMD) were observed among groups in either plane, except that, in the sagittal plane, the malposition group was significantly younger than the proper-placement group (*p* = 0.02). No significant differences were observed in age, sex, or BMD between included and excluded cohorts, but BMI was significantly lower in excluded patients (Supplementary Table S1).

### 3.2. Correlation Between Preoperative Pelvic Parameters and Optimal S2AI Angles

Correlation analysis demonstrated significant relationships between pelvic parameters and optimal S2AI screw trajectories (Table 2). Pelvic tilt (PT) showed a moderate inverse correlation with the optimal transverse angle (OTA) (*r* = −0.51, *p* < 0.001) and a strong inverse correlation with the optimal sagittal angle (OSA) (*r* = −0.79, *p* < 0.001). Pelvic incidence (PI) was also inversely correlated with both OTA (*r* = −0.64, *p* < 0.001) and OSA (*r* = −0.38, *p* < 0.001). Sacral slope (SS) demonstrated a weaker inverse correlation with OTA (*r* = −0.41, *p* < 0.001) and was not significantly correlated with OSA (*r* = 0.16, *p* = 0.19).

### 3.3. Direction-Specific Generalized Estimating Equations (GEE) Analysis of Trajectory Deviation and Screw Malposition

In the axial plane, both lateral and medial deviation magnitudes were significantly associated with their respective cortical violations (Table 3). Greater lateral deviation was associated with increased odds of lateral violation (β = 0.85, SE = 0.22, OR 2.33, 95% CI 1.51–3.59, *p* < 0.001). Similarly, greater medial deviation was significantly associated with increased odds of medial violation (β = 0.74, SE = 0.21, OR 2.10, 95% CI 1.40–3.15, *p* < 0.001).

In the sagittal plane, greater inferior deviation was significantly associated with inferior violation (β = 0.33, SE = 0.09, OR 1.39, 95% CI 1.18–1.65, *p* < 0.001), whereas superior deviation was not significantly associated with violation.

Among all cortical violations, only one case of medial violation in the axial plane was associated with vascular injury. No neurological injury, sacroiliac joint complication, postoperative symptoms due to screw malposition, or revision surgery attributable to screw malposition were observed among the remaining cortical violation cases in either plane.

### 3.4. Safety Margin Determination Using ROC Analysis

**Figure 2:**
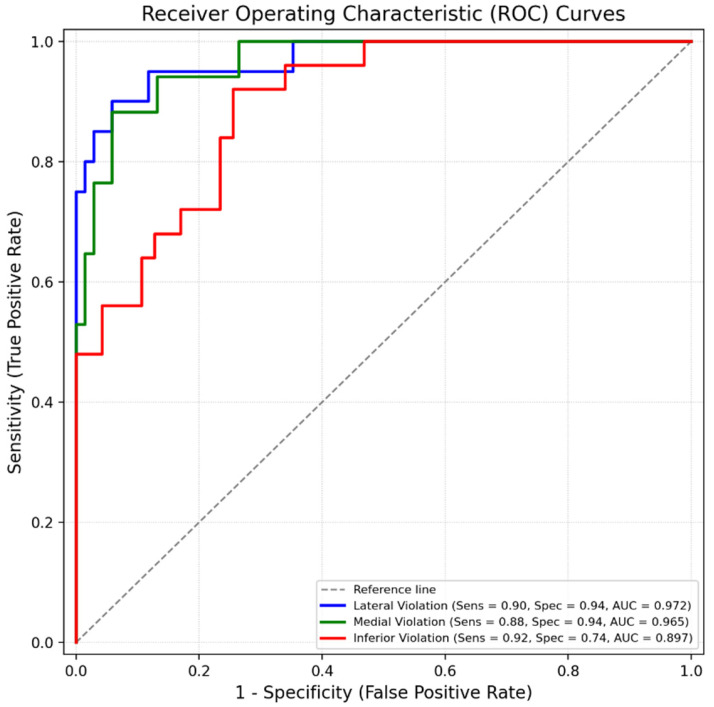
Receiver Operating Characteristic (ROC) curve analysis for predicting cortical violation based on trajectory deviation from the patient-specific optimal S2AI screw angle. ROC curves were constructed to evaluate the predictive performance of deviation magnitude in each direction. In the axial plane, cutoff values of 1.5° for lateral deviation and 8.1° for medial deviation were identified. In the sagittal plane, a cutoff value of 18.5° was identified for inferior deviation. The area under the curve (AUC) was 0.972 for lateral, 0.965 for medial deviations and 0.897 for inferior deviation, indicating high predictive accuracy. No meaningful cutoff was identified for superior deviation. The x-axis represents 1—specificity, and the y-axis represents sensitivity.

ROC curve analysis was performed to determine cutoff values of trajectory deviation associated with screw malposition (Figure 2). The optimal cutoff values were 1.5° lateral (Sensitivity = 0.900, Specificity = 0.941, AUC = 0.972, *p* < 0.001) and 8.1° medial (Sensitivity = 0.882, Specificity = 0.941, AUC = 0.965, *p* < 0.001) from the OTA, 18.5° inferior (Sensitivity = 0.920, Specificity = 0.744, AUC = 0.897, *p* < 0.001) from the OSA, for good predictive ability of screw violations. No meaningful cutoff was identified for superior deviation due to the lack of significant association.

### 3.5. Reliability

Interobserver reliability for measurement of optimal and actual screw angles were high (Table 4). In the axial plane, the intraclass correlation coefficient (ICC) was 0.996 for OTA and 0.798 for actual screw angle. In the sagittal plane, ICC values were 0.946 for OSA and 0.896 for actual screw angle. All measurements demonstrated statistically significant agreement (*p* < 0.001).

## 4. Discussion

Accurate sacropelvic fixation remains technically challenging because the lumbosacral junction is exposed to high biomechanical stress and complex anatomy [3,4]. These features contribute to a higher rate of sacropelvic fixation failure [1,2]. For this reason, reliable sacropelvic fixation techniques are essential for achieving durable fusion in long spinal constructs. Among the available methods, S2-alar-iliac (S2AI) screw fixation has become widely adopted due to several advantages compared with traditional iliac screws [6,7,8,9,11,18]. Previous anatomical and radiological studies have primarily focused on identifying the ideal entry point and trajectory for S2AI screw placement [5,6,12,19,20]. However, in actual surgical practice—particularly when using fluoroscopy-assisted free-hand techniques—surgeons rarely reproduce these exact angles. Therefore, rather than focusing solely on ideal trajectories, S2AI screw placement may be better understood as a process that accommodates a degree of trajectory deviation in clinical practice.

This study quantified direction-specific safety margins around patient-specific optimal S2AI screw trajectories and demonstrated that trajectory deviation is a direction-dependent predictor of cortical violation.

**Figure 3:**
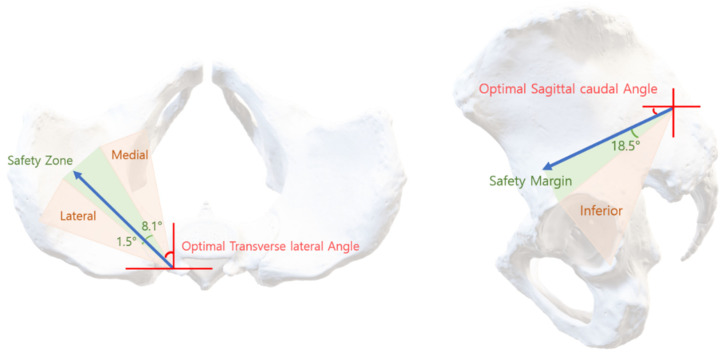
Conceptual model illustrating the asymmetric safety corridor around the optimal S2AI trajectory. The red angle represents the optimal trajectory angle. The green zone indicates the allowable safety margins derived from ROC analysis, while yellow zone represents trajectories associated with cortical violation. In the axial plane, the safe corridor is asymmetric, with substantially greater tolerance for medial deviation than lateral deviation. In the sagittal plane, excessive inferior deviation beyond the defined safety margin increases the risk of violation. This figure is a schematic illustration intended to demonstrate the concept of probabilistic direction-specific safety corridors and is not derived from direct patient imaging. The angular proportions displayed are not drawn to scale and are intended solely for conceptual visualization.

In the axial plane, both medial and lateral deviations were significantly associated with cortical violation, although the tolerance differed substantially by direction. Lateral deviation showed a steep increase in risk, with each 1° increment associated with more than a twofold increase in the odds of violation, whereas medial deviation demonstrated a comparatively wider permissible range. ROC analysis further supported this asymmetry, identifying a markedly narrower cutoff for lateral deviation than for medial deviation (Figure 2). These findings suggest that the safe intraosseous corridor is not centered symmetrically around the optimal trajectory but is instead bounded by direction-specific anatomical constraints (Figure 3).

In contrast, in the sagittal plane, only inferior deviation was significantly associated with cortical violation, while superior deviation did not reach statistical significance. This finding should be interpreted with caution. Anatomically, the relatively large inferior tolerance may reflect the geometry of the iliac corridor, which provides a longer cancellous pathway before inferior cortical breach occurs. However, the number of superior violation cases was limited, which may have reduced statistical power to detect an association. Therefore, while the data support a clinically relevant threshold for inferior deviation, the absence of a significant association for superior deviation should not be interpreted as evidence of safety in that direction. Future studies with larger sample sizes are required to better characterize sagittal plane tolerance in both directions.

The observed asymmetry in safety margins may be explained by the anatomical configuration of the ilium. The iliac corridor has a medially concave geometry, allowing greater tolerance for medial deviation, whereas lateral deviation more rapidly approaches the cortical boundary. From a surgical perspective, these findings suggest that avoiding lateral deviation is particularly important during free-hand insertion. Medial cortical violation may place adjacent neurovascular structures at risk, whereas lateral violation more commonly involves the iliac cortex and surrounding soft tissues. Given the inherent variability of radiographic measurements and fluoroscopic assessment, the identified 1.5° lateral cutoff should be interpreted as a probabilistic indicator of increased risk rather than a precise intraoperative target, reflecting the narrow anatomical tolerance for lateral deviation.

**Figure 4:**
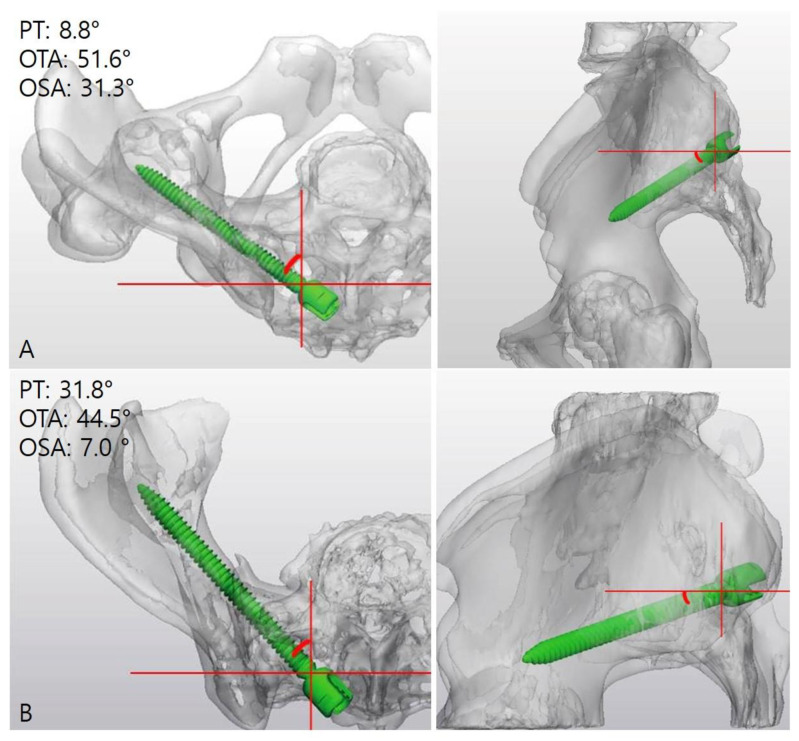
Influence of Pelvic Tilt on Optimal S2AI Screw Angles. (**A**) Axial and sagittal views of screw angle in low pelvic tilt patient, showing relatively greater optimal angles in each plane; (**B**) Axial and sagittal views of screw angle in high pelvic tilt patient, depicting relatively smaller optimal angles in each plane. The corresponding PT, OTA, and OSA values are displayed within each panel.

Our results also confirm that pelvic parameters significantly influence optimal S2AI trajectories. Pelvic tilt demonstrated strong inverse correlations with both optimal transverse and sagittal screw angles (Figure 4). These findings are consistent with previous studies suggesting that pelvic morphology affects the orientation of the S2AI corridor [15,16,21]. The strong relationship between pelvic tilt and sagittal screw angle supports the concept that optimal S2AI trajectories should be individualized according to patient-specific pelvic alignment rather than applied as fixed angular values.

This study has several important limitations. First, the retrospective design introduces the potential for selection bias. Sagittal plane analysis was limited by incomplete CT coverage in earlier cases, resulting in a reduced sample size. BMI was significantly lower in excluded patients, suggesting residual selection bias. Furthermore, only four superior cortical violations were identified, limiting statistical power for analyses of superior deviation. Because these exclusions were more common in earlier cases, temporal and technical factors may have influenced the results. These exclusions may have influenced the observed distribution of trajectory deviations and, consequently, the generalizability of the reported ROC-derived cutoff values. Second, the outcome measure was based on radiographic cortical violation rather than clinical complications. Although cortical breach is a relevant surrogate marker of technical accuracy, it does not directly reflect neurovascular injury or clinical outcomes. In this cohort, clinically significant complications related to screw malposition were rare, limiting our ability to evaluate the relationship between radiographic breach and clinical outcomes. Third, the GEE analyses were performed without multivariable adjustment. Because the number of malposition events was relatively limited, particularly in the sagittal plane, inclusion of multiple covariates could have resulted in model overfitting and unstable estimates. Although the primary objective was to evaluate the direct association between trajectory deviation and cortical violation, potential confounding effects of factors such as sex, pelvic morphology, and bone quality cannot be excluded. Future studies with larger cohorts may allow more comprehensive multivariable modeling. Fourth, pelvic parameters were measured using CT scout images obtained in the supine position, which may differ from functional alignment in standing posture. In particular, pelvic tilt and sacral slope may vary between supine and standing positions because of postural compensation and weight-bearing effects. Therefore, caution is warranted when directly extrapolating these measurements to standing spinopelvic alignment. Finally, this was a single-center study with a predominantly Asian population. Previous studies have suggested that sex and ethnicity can influence pelvic morphology [14,19,22], which may limit generalizability to other populations with different pelvic morphologies. Additionally, the reported cutoff values may vary with different implant dimensions and should not be assumed to be directly generalizable to all implant systems.

Despite these limitations, this study provides clinically relevant insights by translating theoretical optimal trajectories into practical tolerance ranges applicable to fluoroscopy-assisted free-hand techniques. By quantifying direction-specific safety margins, the present findings may assist surgeons in making intraoperative adjustments when exact reproduction of the optimal trajectory is not feasible. Importantly, these margins should be interpreted as probabilistic thresholds rather than absolute boundaries, and they should be applied in conjunction with anatomical judgment and intraoperative imaging.

## 5. Conclusions

S2-alar-iliac screw placement can be understood as a tolerance-based process centered on a patient-specific optimal trajectory. This study demonstrates that the allowable deviation from this optimal trajectory is direction-dependent and asymmetric, particularly in the axial plane, where lateral deviation is associated with a substantially narrower safety margin than medial deviation. In the sagittal plane, a clinically relevant threshold was identified for inferior deviation, whereas the tolerance for superior deviation remains uncertain.

These findings provide a quantitative framework for understanding acceptable trajectory deviation during fluoroscopy-assisted free-hand S2AI screw placement. Rather than relying solely on ideal angular targets, surgeons may benefit from incorporating direction-specific tolerance ranges into intraoperative decision-making. Further prospective and multicenter studies are warranted to validate these safety margins and to correlate them with clinical outcomes.

## Figures and Tables

**Table 1:** Baseline preoperative characteristics of the patients in proper and malposition group for axial and sagittal plane.

Plane	Screw Position	Age (Years)	BMI (kg/m^2^)	BMD	Female, N (%)
Axial	Proper (N = 68)	68.04 (4.85)	25.78 (3.39)	−1.88 (0.95)	61 (89.7)
	Lateral (N = 20)	68.30 (5.29)	26.00 (3.50)	−2.01 (0.89)	20 (100)
	Medial (N = 17)	69.53 (4.85)	25.83 (3.02)	−1.99 (0.83)	16 (94.1)
	*p*-value	0.54	0.97	0.80	0.30
Sagittal	Proper (N = 47)	69.40 (3.70)	25.21 (2.93)	−1.93 (0.85)	40 (85.1)
	Superior (N = 4)	69.00 (5.94)	25.84 (4.10)	−2.73 (0.64)	4 (100.0)
	Inferior (N = 25)	66.68 (3.78)	26.35 (3.97)	−2.07 (0.84)	25 (100.0)
	*p*-value	0.02 *	0.39	0.19	0.09

All analyses in Table 1 were performed at the screw level. Patient-level demographic variables were assigned to individual screws for screw-level comparison. Values are presented as mean ± standard deviation or number (%). Abbreviations: BMI, Body mass index; BMD, Bone mineral density; N, Numbers presented at screw levels. * Statistical significance was defined as *p* < 0.05.

**Table 2:** Correlation of pelvic parameters to optimal screw angles in axial and sagittal plane.

Pelvic Parameters	OTA		OSA	
	*r*	*p*-Value	*r*	*p*-Value
PT	−0.51	<0.001 *	−0.79	<0.001 *
SS	−0.41	<0.001 *	0.16	0.19
PI	−0.64	<0.001 *	−0.38	<0.01 *

Abbreviations: OTA, Optimal transverse lateral angle; OSA, Optimal sagittal caudal angle; PT, Pelvic tilt; SS, Sacral slope; PI, Pelvic incidence; *r*, Pearson correlation coefficient. * Statistical significance was defined as *p* < 0.05.

**Table 3:** Direction-specific Generalized Estimating Equations (GEE) analysis of trajectory deviation and screw malposition.

Plane	Malposition	β	SE	OR (95% CI)	*p*-Value
Axial	Lateral	0.85	0.22	2.33 (1.51–3.59)	<0.001 *
	Medial	0.74	0.21	2.10 (1.40–3.15)	<0.001 *
Sagittal	Superior	−0.03	0.07	0.97 (0.84–1.11)	0.64
	Inferior	0.33	0.09	1.39 (1.18–1.65)	<0.001 *

Abbreviations: β, Coefficient; SE, Standard error; OR, Odds ratio; CI, Confidence interval. * Statistical significance was defined as *p* < 0.05.

**Table 4:** ICCs between reviewers.

Measurement		ICC (95% CI)	Mean Interobserver Angular Difference (°)	*p*-Value
Axial plane	OTA	0.996 (0.994–0.998)	0.099	<0.001 *
	Actual screw angle	0.798 (0.661–0.884)	3.748	<0.001 *
Sagittal plane	OSA	0.946 (0.919–0.965)	0.400	<0.001 *
	Actual screw angle	0.896 (0.817–0.942)	3.282	<0.001 *

Reliability was assessed using the intraclass correlation coefficient (ICC) based on a two-way random-effects model for absolute agreement. ICC values were interpreted as follows: >0.90, excellent; 0.75–0.90, good; 0.50–0.75, moderate; and <0.50, poor. All evaluated parameters demonstrated good to excellent reliability, confirming the robustness of the radiographic analysis. Abbreviations: ICC, Intraclass correlation coefficients; OTA, Optimal transverse lateral angle; OSA, Optimal sagittal caudal angle. * Statistical significance was defined as *p* < 0.05.

## Data Availability

The data presented in this study are available on request from the corresponding author. The data are not publicly available due to privacy and ethical restrictions regarding patient medical records.

## Supplementary Materials

The following supporting information can be downloaded at: https://www.mdpi.com/article/10.3390/jcm15124495/s1, Table S1: Baseline preoperative characteristics of the patients included and excluded in analysis for axial and sagittal plane.
