# Supplementary material for: Asymmetric Safety Corridors for Free-Hand S2-Alar-Iliac Screw Placement: Quantifying Direction-Specific Tolerance Around Patient-Specific Optimal Trajectories
Source: J Clin Med. 2026 Jun 10;15(12):4495. doi: 10.3390/jcm15124495 (PMC13302756; doi:10.3390/jcm15124495)
Supplement: Supplementary file 1 [file jcm-15-04495-s001.zip › jcm-4289894-supplementary.pdf]

Supplementary Table S1. Baseline preoperative characteristics of the patients included and excluded in analysis for axial and sagittal plane.

Plane	Screw Position	Age (years)	BMI (kg/m^2)	BMD	Female, N (%)
Axial	Included (N=62)	68.24 (5.08)	25.67 (3.27)	-1.92 (0.91)	57 (91.9)
	Excluded (N=28)	69.64 (4.56)	23.20 (2.36)	-2.09 (0.54)	23 (82.1)
	p-value	0.20	<0.001*	0.29	0.31
Sagittal	Included (N=41)	68.27 (4.07)	25.65 (3.40)	-1.99 (0.88)	37 (90.2)
	Excluded (N=49)	69.02 (5.58)	24.27 (2.94)	-1.96(0.76)	43(87.8)
	p-value	0.46	0.04*	0.86	0.71

All analyses in Supplementary Table S1 were performed at the patient level. Values are presented as mean ± standard deviation or number (%). Abbreviations: BMI, Body mass index; BMD, Bone mineral density; N, Numbers presented at patient levels.

* Statistical significance was defined as $p < 0.05$.
